# A Contact-Sensitive Probe for Biomedical Optics

**DOI:** 10.3390/s22062361

**Published:** 2022-03-18

**Authors:** Marco Renna, Adriano Peruch, John Sunwoo, Zachary Starkweather, Alyssa Martin, Maria Angela Franceschini

**Affiliations:** 1 Optics at Athinoula A. Martinos Center for Biomedical Imaging, Massachusetts General Hospital, Harvard Medical School, Boston, MA 02115, USA; adriano.peruch@gmail.com (A.P.); jsunwoo@mgh.harvard.edu (J.S.); zstarkweather@mgh.harvard.edu (Z.S.); amartin46@mgh.harvard.edu (A.M.); mfranceschini@mgh.harvard.edu (M.A.F.); 2 Department of Radiology, Harvard Medical School, Boston, MA 02115, USA

**Keywords:** contact, capacitive, proximity sensing, probe, NIRS, DCS, biomedical, optics, continuous monitoring, laser safety

## Abstract

Capacitive proximity sensing is widespread in our everyday life, but no sensor for biomedical optics takes advantage of this technology to monitor the probe attachment to the subject’s skin. In particular, when using optical monitoring devices, the capability to quantitatively measure the probe contact can significantly improve data quality and ensure the subject’s safety. We present a custom novel optical probe based on a flexible printed circuit board which integrates a capacitive contact sensor, 3D-printed optic fiber holders and an accelerometer sensor. The device can be effectively adopted during continuous monitoring optical measurements to detect contact quality, motion artifacts, probe detachment and ensure optimal signal quality.

## 1. Introduction

The interest towards non-invasive optical probing of human tissues in depth has constantly increased in recent years, with near-infrared optical spectroscopy techniques finally reaching both the clinical and commercial fields [1,2]. Diffuse optics systems for human measurements rely on bright light sources and extremely sensitive photodetectors to characterize the tissue under test. Proper sensor attachment to the skin is pivotal for most biomedical devices, and becomes critical for near infrared spectroscopy (NIRS) systems. Poor coupling between the optical probe and the subject’s skin may lead to direct light leakages and motion artifacts [3], thus corrupting the acquired data [4] and potentially posing a risk to the subject’s eye safety in the case of a complete probe detachment. This is particularly relevant for diffuse correlation spectroscopy (DCS), a NIRS method which employs long coherence-length lasers at a power above the ANSI eye safety limits [5,6].

Optical probes for fiber-based NIRS systems are typically made of flexible 3D-printed materials that incorporate the injection and detection fibers and accommodate for curved surfaces [7,8,9]. Systems with a limited number of channels tend to rely on external supports to guarantee proper probe attachment, such as bandages or elastic/adhesive straps tightened around the probe. Alternatively, systems targeting full-head coverage tend to rely on custom caps or helmets to secure the optodes [10,11,12], occasionally making use of spring-loaded optical systems to maximize the light collection through different types of hair [12]. Despite extensive efforts to improve the optical coupling, none of the custom probes and optodes presented in the literature provide a direct measurement of the sensor attachment to the skin. Thus, the quality of the acquired optical data cannot be ensured easily and can be verified only during signal post-processing. Additionally, some applications may prevent the use of external supports to secure the optical probe to the subject, and the use of bright light sources can pose serious eye safety issues when the subject’s blinking reflex is impaired or the wavelength used is in the infrared range, hence not visible. These limitations are common when performing optical measurements on infants during the first days of life or on unconscious patients, and may hinder the usability of NIRS devices in clinical continuous monitoring applications.

Capacitive sensing is a well-known proximity measurement technique that is widespread in our everyday life and that relies on measuring the capacitance variation induced by capacitive coupling between electrically conductive electrodes and an object in their proximity [13,14]. The object introduces a variation in the dielectric constant near the sensing electrode, causing a variation in the electrode capacitance that can be measured by a dedicated network [15]. In addition to smart environments [16] and human–computer interface [17], capacitive proximity sensing has been effectively adopted in a variety of applications such as non-destructive evaluation of low-conductivity materials [18], 3D-imaging [19], and interface for interaction with plants [20]. The sensing electrode is usually made by a patch of conductive material on a non-conductive substrate. Thus, capacitive proximity sensors are often manufactured into printed circuit boards (PCBs), which integrate the readout circuit and tailor the sensor to specific applications, such as: human touch interface, water content measurement in paper pulp [21], air bubble detection in fluidic flow [22], or aerodynamic pressure measurements [23]. Several devices are based on flexible PCBs, as is the case of human touch [24,25] and grip sensors [26], and tactile sensing arrays for robots [27]. This allows for the minimization of bulk and the customization of the sensor shape for applications where adapting to the sensing surface is crucial. Flexible PCBs are a low-cost commercially available manufacturing technology which show ideal features for the design of an optical probe, as they integrate capacitive electrodes and additional sensors while offering a sturdy support for the optical fiber housings, with the PCB being the body of the probe itself. The substrate flexibility and the customizable shape and thickness allow the probe to be tailored to the specific application and maximize the fitting to various body shapes. The main drawbacks of PCB-based capacitive sensing are its high sensitivity to noise, stray capacitance, and the environmental conditions such as temperature and humidity.

Here, we present a novel sensor based on capacitive proximity sensing, which can be integrated into optical probes to continuously monitor the contact with the subject and promptly alert users/operators in case of an unanticipated detachment, ensuring patient safety and data quality, especially in continuous monitoring applications. The contact sensor is based on a capacitive sensitive electrode integrated in a flexible PCB, and commercially available driving and readout integrated circuit (IC). Human contact with the probe adds a macroscopic amount of additional capacitance to the sensing network, and variation in the overall sensing network capacitance can be detected by the readout electronics. By periodically measuring the probe capacitance, it is possible to constantly monitor the probe attachment to the skin and output a contact quality signal in real time. An additional accelerometer sensor integrated on the probe PCB allows for the detection of motion artifacts in the acquired optical data [28]. The presented instrument has been extensively validated and successfully adopted during continuous NIRS-DCS monitoring on premature-born infants in a clinical setting.

## 2. Materials and Methods

The fully custom instrument is based on: (i) a flexible probe-shaped PCB which hosts a contact-sensing electrode, an accelerometer sensor, and the 3D-printed fiber holders for diffuse optics measurements; (ii) a control board PCB with the acquisition electronics. The control board is manufactured on standard FR4 substrate and is battery-operated to minimize the hazard-risk in clinical environments.

### 2.1. Sensing Electrode Shape

**Figure 1:**
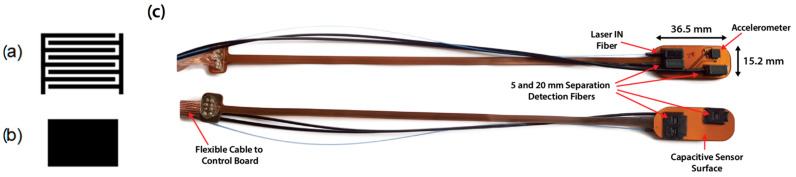
(**a**) Drawing of an interdigitated comb electrode (V1). Two comb-shaped electrically conductive electrodes are placed in an interdigitated fashion and each comb finger acts as a plate of a mutual planar capacitor with the adjacent fingers. (**b**) Drawing of a self-capacitance single electrode (V2), where an isolated copper plate acts as a plate of a planar capacitor. (**c**) Pictures of the probe flexible printed circuit board (PCB) equipped with the single-electrode contact sensor. Top—Probe top view: the accelerometer Integrated Circuit (IC) and the 3D-printed fiber holders, not in contact with the subject’s skin. Bottom—Probe bottom view: a copper plane covering the whole bottom layer of the PCB acts as single sensing electrode for the contact measurement.

Several electrode shapes for capacitive sensing are present in the literature, consisting of different sizes, performance capabilities, and operating principles [14], with the most common designs for human interface being: (i) round or rectangular solid areas, implementing a self-capacitance sensor; (ii) interdigitated comb or key electrodes, implementing mutual capacitance configurations [29]. In this design, we evaluated two different electrode shapes: the mutual-capacitance interdigitated comb electrode (V1, Figure 1a) and the self-capacitance single electrode (V2, Figure 1b).

In the interdigitated comb electrode, the contact measurement takes advantage of the fringing electric field between two adjacent copper strips, which act as the two plates of a planar capacitor [30]. Any material put in contact/proximity with the electrodes changes the dielectric constant of the media between the two capacitor plates, resulting in a capacitance variation in the electrode. Since the sensitive area is at a maximum between the two adjacent strips, contact applications usually adopt an interdigitated comb-shape to maximize the sensitivity of the sensing surface [14,29]. The width of each strip and the separation between adjacent strips can be adjusted to tailor the sensor performance to the specific application [14].

The single-electrode sensor relies on the same fringing capacitance effect of the parallel strips but makes use of a single plate of a planar capacitor and the object in its proximity acts as grounded secondary plate [15].

The mutual-capacitance sensor is made of two interdigitated comb electrodes, each one made of 300 μm copper strips with 1.4 mm pitch, leading to a 400 μm spacing between two adjacent strips. The electrode of the self-capacitance sensor covers all the available surface of the PCB.

### 2.2. Probe Design

The presented flexible probe PCB (pictures in Figure 1c) is based on a flexible polyimide substrate (0.1 mm thickness) which allows the probe to perfectly adhere to the skin. The sensor probe allows for three simultaneous measurements: (i) contact-sensing; (ii) 3-axis acceleration sensing for motion detection; (iii) light injection and detection for DCS acquisition at multiple source-detector separations. The contact sensor is a copper pattern on the bottom layer of the PCB (35 μm copper thickness), and a copper plane (35 μm thickness) on the top layer of the PCB is connected to ground, acting as passive shield for the sensing electrode [30] to minimize the sensitivity to unwanted contacts from the top side of the probe.

Mounted on the top layer of the sensor probe PCB, a compact and low-power 3-axis MEMS accelerometer IC (ADXL327 from Analog Devices Inc., Norwood, MA, USA) allows the probe movement to be detected and motion artifacts in the acquired data to be identified. The sensor measures the acceleration along three axes with a full-scale range of ±2 g and a sensitivity of 355 mV/g (g = 9.8 m/s^2^) when powered at 2.5 V, with 1.6 kHz bandwidth, and provides three independent analog outputs. To further reduce risks of hazard related to electronic equipment, the maximum current that can be drawn from the accelerometer dedicated 2.5 V power supply is limited to 30 mA.

Finally, two 3D-printed fiber holders are mounted into two apertures of the PCB for light injection and detection at two source detector separations: 5 and 20 mm. A circular (3.5 mm diameter) holographic diffuser with 40° diffusing angle is integrated with the injection fiber holder to increase the surface area illuminated by the injection fiber.

The sensing area for the comb-electrode probe is equal to 44 × 1 m^2^ and 36.5 × 15.2 mm^2^ for the single-electrode sensor, and a narrow 3.5 × 200 mm^2^ PCB strip distances the probe sensing area from the cable connection. A 1.5 m extremely flexible flat cable connects the probe to the control board, minimizing the bulk on the subject side and guaranteeing optimal flexibility in probe positioning. It is important to note that the dimensions of the sensing electrodes presented in this work are determined by the probe optical geometry, and sensors with a smaller sensitive surface can still guarantee sufficient performance if probe miniaturization is needed.

### 2.3. Contact-Sensing Circuit and Control Board Design

**Figure 2:**
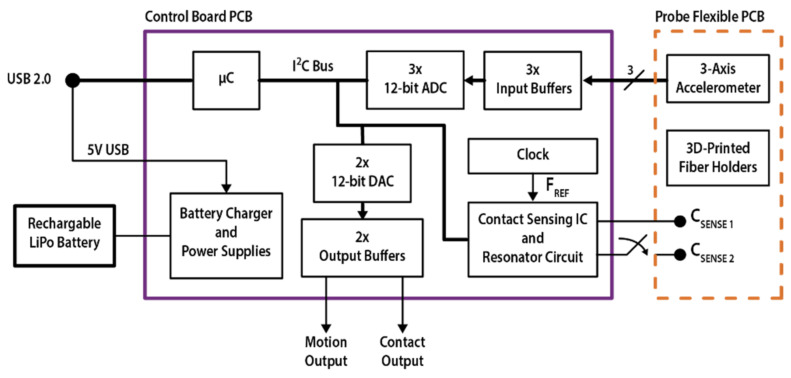
Simplified block diagram of the presented instrument. The optical probe is based on a flexible PCB which incorporates, as well as 3D-printed fiber holders for optical spectroscopy measurements, a 3-axis MEMS accelerometer and an integrated planar capacitive sensor for contact detection. An extremely flexible flat cable connects the probe to the control board where the contact and accelerometer signals are acquired through dedicated front-end circuitry. A microcontroller runs the measurement routine and provides the acquired signal either as digital output through a USB 2.0 link to the external computer or as an analog output through two dedicated signal chains and BNC connectors, allowing for the integration of the device in different measurement setups. A battery management integrated circuit allows for battery operation, reducing the bulk and the hazard risk at the same time, and fostering the adoption of the instrument in clinical environments.

A simplified block diagram of the instrument is shown in Figure 2. The capacitive sensor readout is based on a commercially available IC (FDC2212 from Texas Instruments [31]) and a LC resonant network made of a fixed LC resonant oscillator on the control board and the variable capacitor on the sensor probe. The contact-sensing IC excites the resonant network, measures the frequency of the oscillation (F_SENS_) as a ratio with respect to an external reference clock source (see F_REF_ in Figure 2) and converts it into a 28-bit value, with the digital output equal to (F_SENS_/F_REF_) × 2^28^. F_REF_ in the presented design is 40 MHz, as this value allows for a wide capacitance measurement range and the finest resolution allowed by the sensing IC, i.e., 40 MHz / 2^28^ = 0.149 Hz. From the measured oscillation frequency of the resonant network, it is possible to retrieve the total capacitance C by reversing the formula F_SENS_ = 1/2π(LC)^1/2^ [30,31], where L is the inductance value of the tank inductor (15 μH in this design). The resonant network was designed in order to support the two different electrode shapes presented in this work, the interdigitated comb electrodes (V1) and single electrode (V2), according to the manufacturer datasheet [31].

The three accelerometer signals are independently buffered, low-pass filtered, and then sampled through a 3-channel Analog-to-Digital Converter (see ADC in Figure 2).

A microcontroller manages the communication bus, acquiring the accelerometer and contact signals and running the measurement routine. A button interfaces the device with the user and triggers the acquisition of a baseline value for the movement and contact measurements, storing: (i) the output of the contact-sensing IC proportional to the measured oscillation frequency of the resonant network; (ii) the 3 accelerometer signals, proportional to the acceleration measured on the 3-reference axis of the device. The modulus of the variations in the three accelerations are calculated with respect to baseline values and are summed into a single value, the motion signal. The variation with respect to the baseline value is also calculated for the frequency (thus the capacitance) measured by the contact sensor to obtain the contact signal. These two digital values are converted into analog signals by a two-channel 12-bit Digital-to-Analog Converter, buffered through a dual-channel high-current OP-AMP with 0–2.5 V output range and provided at instrument output through two standard BNC connectors. This easily integrates the instrument as a stand-alone device in existing measurement setups. Once the button is pressed, the contact analog output is initialized to 1.25 V (VDD/2) and is then driven with a signal directly proportional to the variation in the sensor oscillation frequency from the baseline: a reduction in the overall capacitance on the network (from contact to no contact) results in higher oscillation frequency, and subsequently results in a positive variation in the output. Inversely, when contact is applied on the sensing electrode, it will cause a reduction in the oscillation frequency and a lower output value. The motion analog signal is initialized at 0 V and any positive variation in the signal from the baseline implies a movement detected by the device, whereas a steady difference from the baseline implies a different probe angle.

The microcontroller also communicates with an external PC through a USB 2.0 link, used for instrument debugging, testing and signal acquisition during experimental characterization. A specific LabVIEW (National Instruments) software interface was designed for data acquisition and to adjust the measurement parameters of the contact-sensing network (not shown). The overall data sampling and output rate is 54 Hz during normal operation with analog output.

Finally, a battery management IC operates the instrument from a standard 3.7 V Lithium-Polymer (LiPo) rechargeable battery, and the USB 5 V DC power supply input can be used to recharge it, even during device operation. The adopted LiPo battery features an integrated short-circuit protection and provides up to 600 mAh charge, which leads to a continuous acquisition longer than 24 h. The control board and battery are enclosed into a compact plastic box (dimensions: 9 × 7 × 5 cm^3^) and a panel LED is used to inform the user about the battery level and the baseline acquisition through the button interface.

## 3. Experimental Validation

We performed an experimental validation of the contact-sensing circuit and the two designed sensor shapes. We first tested the contact sensor with and without a hydrogel layer (25 mil thickness, AG6525, MBK Tape Solutions, Chatsworth, CA, USA) on the sensing surface to validate its performance and applicability in a clinical environment. After an extensive validation of the device in time-varying ambient conditions, we adopted the presented flexible probe with integrated motion and contact sensing in a clinical measurement campaign with diffuse optics on extremely low gestational age (ELGA, <29 weeks GA) babies in a neonatal intensive care unit (NICU).

### 3.1. Contact Sensor Validation

We validated the performance of the capacitive sensing network by initially testing the capability of the system in measuring the value of a known capacitance added to the resonant network. For this test, an oscilloscope passive probe (Tektronix P6501R) with 12 pF nominal capacitance was placed in contact with the two resonant network sensing nodes (C_SENSE 1_ and C_SENSE 2_) on two test points on the control PCB, for the two probes V1 and V2. The ground of the oscilloscope probe was shorted to the control PCB ground.

After a preliminary validation, we evaluated the measured capacitance by the probes V1 and V2 when put in direct contact with the skin on the forearm of a healthy volunteer. To minimize the error due to probe positioning, the probe was secured to a vertical translational stage and moved vertically from non-contact to contact. The travel distance of the translational stage and the forearm position were kept constant throughout the measurements.

In all the measurements presented hereafter, a cable length equal to 1.5 m was used to connect the contact probe to the control PCB.

### 3.2. Measurement Stability vs. Ambient Temperature

We experimentally characterized the measurement stability of the device against an 8 °C ambient temperature variation. The probe PCB was enclosed in a box with a heating system with no contact, while the control board was kept at a constant ambient temperature. The temperature was gradually increased in 30 s and was then allowed to return to the baseline.

### 3.3. Measurement Stability vs. Ambient Humidity

We evaluated the device stability and contact-sensing capabilities against ambient relative humidity variations. The probe PCB was enclosed in a box with a humidifier, and no contact was applied on the sensing electrode. The ambient relative humidity was increased from 0 to 100% in about 160 s and monitored with a reference device. Once 100% relative humidity was reached, the back of the hand of a healthy volunteer was applied to verify the device capability to detect contact variations. The water used for the humidifier was at ambient temperature to avoid any contribution due to temperature variations within the box.

### 3.4. Clinical Application

Diffuse correlation spectroscopy is a non-invasive neuro-imaging NIRS technique which utilizes long coherence-length laser sources and single photon detectors to measure the micro-vascular cerebral blood flow [5,6]. It takes advantage of multiple photon scattering events in diffusive human tissues to measure a relative blood flow index and is effectively adopted in neuro-monitoring applications on infants [32].

**Figure 3:**
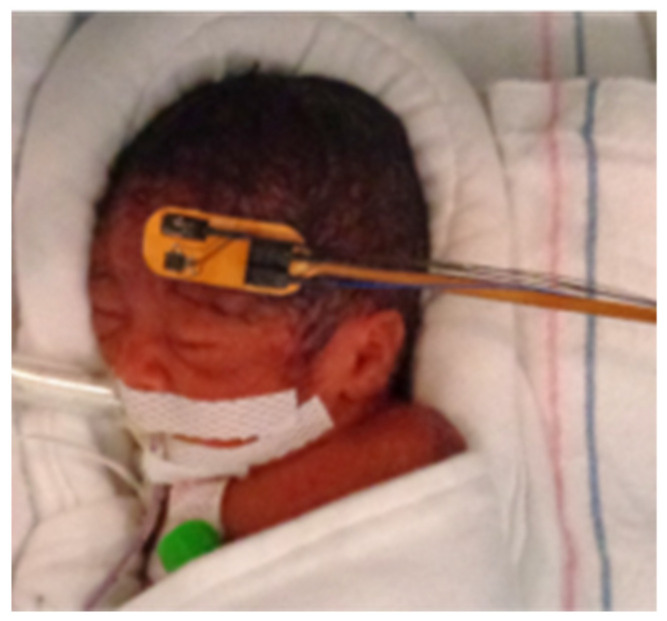
Picture of an extremely preterm infant during a continuous-monitoring diffuse correlation spectroscopy measurement with the presented contact-sensitive optical probe. The measurement was performed in the Giraffe incubator in a neonatal intensive care unit.

After demonstrating the full functionality of the contact-sensing electrode, we adopted the presented system to monitor the attachment of the optical probe on the forehead of ELGA infants during DCS measurements in two different NICU locations at Brigham and Women’s Hospital and Massachusetts General Hospital. The presented device and the proposed measurement protocol were reviewed and approved by the Mass General Brigham institutional review board (IRB) for non-invasive and continuous monitoring of cerebral hemodynamics in extremely low gestational age infants to find association between dysregulation of cerebral blood flow and intraventricular hemorrhage. For these measurements, only the probe V2 with a hydrogel layer was used. The hydrogel layer ensures that the probe is never in direct contact with the delicate infant’s skin to avoid any damage or irritation, while guaranteeing a secure probe positioning. The motion and contact analog output signals were acquired simultaneously with the optical acquisition through the auxiliary inputs of the DCS system used for the measurement [9]. Analog signals were used instead of the USB link due to the limited number of USB serial ports in the computer used in the measurement setup. The contact signal is used to monitor the probe attachment to the subject’s skin and quickly triggers the laser shutdown in case of a detachment, i.e., when the contact signal crosses a user-defined voltage threshold. The motion signal is used to detect motion artifacts in the acquired DCS signals during data analysis. Figure 3 shows a picture of a 26-week gestational age infant inside a Giraffe incubator with the presented flexible probe V2 placed on the forehead during a continuous monitoring multi-distance DCS measurement.

## 4. Results

The steady-state overall capacitance measured by the sensing network with the two probes with/without a hydrogel layer is 279.6/184.1 pF for probe V1 and 161.2/160.7 pF for probe V2. The values of the measured capacitance were calculated in post-processing, starting from the digitized value of the resonant network frequency measured by the contact-sensing IC, assuming all the parameters are constant except for the network capacitance.

**Figure 4:**
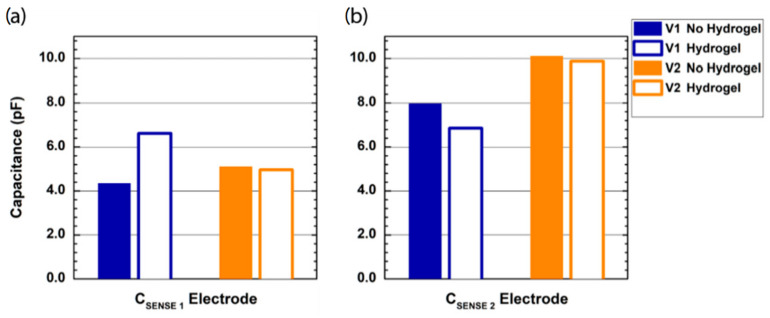
Results of the measurement of the known capacitance of a passive oscilloscope probe (12 pF) on the two sensing nodes ((**a**)—C_SENSE 1_, (**b**)—C_SENSE 2_) of the resonant network on the probe V1 (blue histograms) and V2 (orange histograms). Full/hollow bars report results without/with a hydrogel layer on the capacitive sensor surface. The two graphs share the y-axis label and the legend.

Figure 4 shows the results of the oscilloscope probe capacitance measured by the probes V1 and V2 on the control PCB sensing nodes C_SENSE 1_ (Figure 4a) and C_SENSE 2_ (Figure 4b), with and without a hydrogel layer on the electrode-sensing surface. For the interdigitated comb electrode (probe V1), when no hydrogel is used, the measured capacitance on the node C_SENSE 1_ is slightly higher than 4 pF, whereas the measured capacitance is equal to 8 pF when the oscilloscope probe is applied on the node C_SENSE 2_. Conversely, when a hydrogel layer is used, the measured capacitance on the two electrodes is almost the same, i.e., 6.6 pF on C_SENSE 1_ and 6.8 pF on C_SENSE 2_. Differing from probe V1, for the single electrode probe, the hydrogel layer has a negligible effect on the measured capacitance, whereas there is a strong difference between the values measured on the two sensing nodes, with a measured capacitance of about 5 pF on C_SENSE 1_ and 10 pF on C_SENSE 2_.

**Figure 5:**
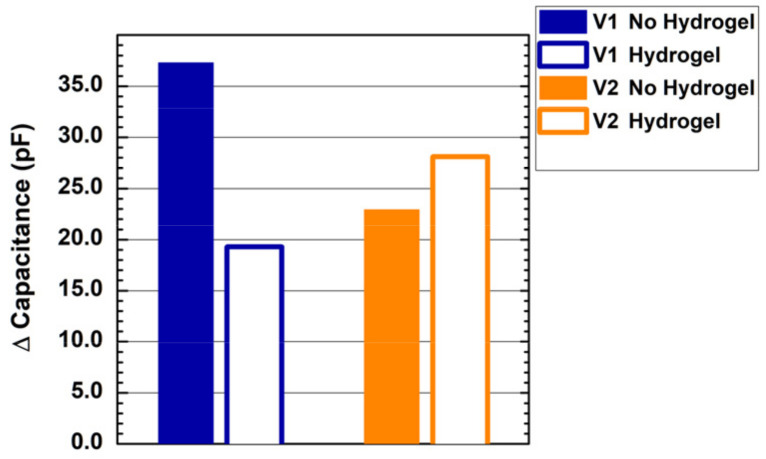
Changes in measured capacitance induced by contact between the sensing electrode and the forearm of a healthy volunteer with the probe V1 (blue histograms) and V2 (orange histograms). Full/hollow bars report results without/with a hydrogel layer on the capacitive sensor surface.

Figure 5 shows the results of the contact measurement on the forearm of a healthy volunteer with the two designed probes with and without a hydrogel layer. We report the measured capacitance variation from steady-state (no contact) to contact. When no hydrogel layer is applied, probe V1 shows better sensitivity, with a measured capacitance of about 37 pF, compared to the 23 pF measured by probe V2. The addition of a hydrogel layer strongly affects the sensitivity of the interdigitated comb electrode on human skin, reducing the measured capacitance to less than 20 pF. Conversely, the hydrogel layer shows a beneficial impact on the sensitivity of the self-capacitance single electrode, with a measured capacitance equal to 28 pF.

**Figure 6:**
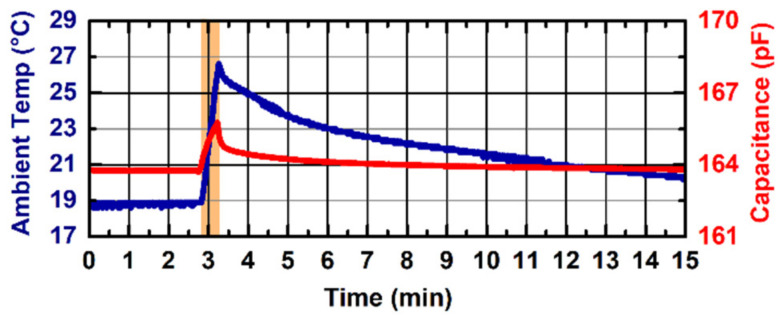
Results of the capacitance stability measurement (red line—right *y*-axis) over 8 °C ambient temperature (blue line—left *y*-axis) variation for the probe V2 with a hydrogel layer and no contact applied to the sensor surface. Ambient temperature is increased from 18.6 to 26.6 °C in 30 s (orange-shaded area) and then let return to baseline.

Figure 6 shows the capacitance measurement stability against ambient temperature variations for the probe V2 with a hydrogel layer applied on the sensing electrode. In this measurement, we report the overall capacitance measured by the contact-sensing circuit. The ambient temperature (blue line, left *y*-axis) varied from 18.6 to 26.6 °C (orange-shaded area), which led to a variation in the overall measured capacitance of 2 pF (red line, right *y*-axis), leading to a thermal drift equal to 0.25 pF/°C. The probe V1 demonstrated a worse performance against ambient temperature variations, with a coefficient of variation equal to 0.55 pF/°C (data not shown). No effect of the hydrogel layer was found during the experiments.

**Figure 7:**
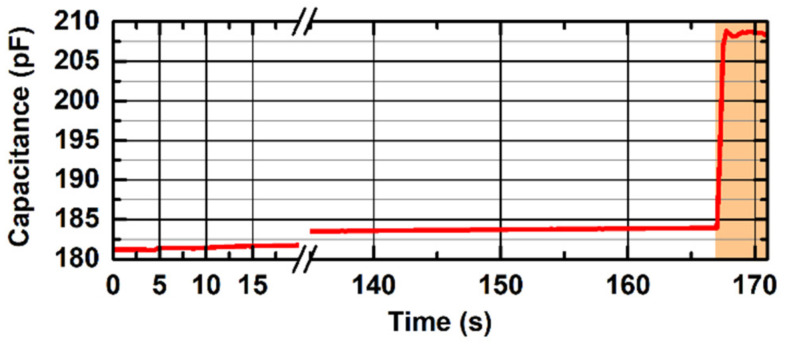
The result of the capacitance stability measurement over ambient relative humidity variation for the probe V2 with a hydrogel layer applied to the sensor surface. During the first 160 s, the ambient relative humidity is increased from <10% to 100% with no contact applied to the sensor surface, resulting in only 2.8 pF variation. After reaching 100% ambient relative humidity, contact with the backhand of a healthy volunteer is applied to the capacitive sensing sensor (orange-shaded area) to demonstrate the device functionality in extreme measurement conditions. The *x*-axis is interrupted between 35 s and 135 s for visualization purposes.

Figure 7 shows the capacitance measurement stability against ambient humidity variations for the probe V2 with a hydrogel layer applied on the sensing electrode. We report the overall capacitance measured by the contact-sensing circuit. During the increase in the ambient relative humidity from 0 to 100% (0 to 160 s), the measured capacitance increases from 181.2 pF to 184.0 pF. Once the relative humidity reached 100%, the backhand of a healthy volunteer was put in contact with the sensing electrode (orange-shaded area) to verify the device functionality in this extreme condition: the induced capacitance variation due to the added contact was equal to 24 pF. No effect of the hydrogel layer was found during the experiments.

### Clinical Validation

**Figure 8:**
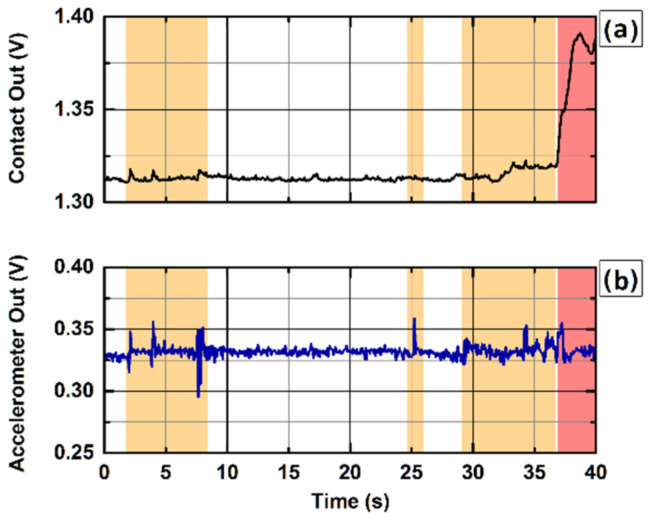
Contact (**a**) and motion (**b**) analog signals acquired with the probe V2 during a diffuse correlation spectroscopy measurement performed on an ELGA baby. It shows a 40 s extract of data acquired while the baby was moving and crying (orange-shaded areas) leading to probe detachment (red-shaded area). Motion artifacts may severely impact the optical data quality, and the capability to identify them during post-processing can be effectively used to mitigate their detrimental effect [4]. The capacitance variation in the sensing electrode induced by baby movements is negligible, whereas a probe detachment results in a macroscopic variation in the contact output analog signal, which can be easily detected by the acquisition instrumentation and used to trigger the laser shutdown and guarantee patient safety.

Figure 8 reports an extract of data acquired during continuous monitoring DCS acquisition on an ELGA baby with the presented contact-sensitive optical probe. The graphs show 40 s of data acquired when the baby was crying and moving (orange-shaded areas), resulting in motion (b) of the optical probe, while the contact (a) of the sensing probe had been maintained, and eventually leading to probe detachment (red-shaded area), as indicated by large signal changes in the contact (a). When the optical probe detaches from the baby’s skin, the contact signal experiences an abrupt macroscopic positive variation, signaling a probe-detachment event, which can be effectively used to immediately trigger the laser shutdown, whereas the variation in the motion signal in correspondence of the probe detachments is undistinguishable from baby movements.

## 5. Discussion

The contact and accelerometer sensor probe for biomedical optics devices presented here allows the user to constantly monitor the probe attachment to the subject’s skin during optical measurements. Proper probe attachment is fundamental to ensure subject eye safety and to acquire quality data. The ability to promptly detect a probe-detachment event and immediately shutdown the light emission can dramatically change the outcome of these events, especially in high-risk applications, such as optical measurements on premature babies or unconscious subjects. While an accelerometer signal can effectively detect motion artifacts in the acquired optical data during post-processing, the contact signal provides additional user safety and data quality metrics in real time.

The two designed electrodes—the interdigitated comb electrode (V1) and the single electrode (V2)—demonstrated strong performance in detecting contact variations on the probe sensitive surface. The single electrode probe proved better operation in terms of skin-contact sensing when a hydrogel layer is applied on the sensor surface, and in terms of thermal stability. Other commercially available adhesive materials (e.g., Mepitel One, Mölnlycke Health Care AB, Gothenburg, Sweden) were tested, with no losses in performance. An overall variation of only 2 pF over 8 °C ambient temperature variation can be negligible compared to the >20 pF capacitance variation experienced when skin is put in contact with the device. Furthermore, the capability to detect contact variations in a 100% relative humidity environment enables the use of the device in high-humidity environments such as Giraffe incubators in NICUs.

The interdigitated comb electrode measured a higher capacitance variation when put in direct contact with the skin, but the addition of a hydrogel layer resulted in a dramatic worsening of the contact-sensing performance. This may be due to limited depth resolution of the interdigitated comb electrode, with the hydrogel layer adhering to the sensor surface resulting in it becoming the main source for the dielectric variation. This is confirmed by the strong difference in steady-state capacitance measured by the sensing IC for the probe V1 with and without hydrogel layer, i.e., 279.6 and 184.1 pF, while the variation experienced by V2 with the addition of the hydrogel layer is negligible. A redesign of the interdigitated comb electrode with increased spacing between adjacent copper strips may help to reduce the crosstalk between the electrodes, and subsequently, the stray capacitance induced by the hydrogel layer.

The capacitance measurement over relative ambient humidity variations for probe V1 demonstrated a performance comparable to that of probe V2, and the measurement over ambient temperature exhibited a similar temporal profile as well, despite a higher sensitivity to temperature variations. Humidity and temperature dependence for the probe V1 are not presented at this time, since this probe was not selected for the clinical application due to worse contact measurement performance in presence of additional adhesive layers. Thus, the probe V1 measurement stability results add limited additional information to the scope of this work.

Finally, it must be noted that the capacitance measurements presented in Figure 4, Figure 5, Figure 6 and Figure 7 include a systemic uncertainty due to the value of the tank inductor (i.e., nominally 15 µH) used to estimate the overall network capacitance. Tolerances for surface-mount multilayer film inductors in small packages (0603 SMD in the presented design) are up to ±20%, and actual values are subject to temperature variations and aging. Finally, in our estimation, we neglected the stray inductance added to the resonant network by the 1.5 m-long wires connecting the probe to the control board. The same sensing network was used to perform all the measurements in Figure 4, Figure 5, Figure 6 and Figure 7 in order to keep the results consistent. This uncertainty is not present in the contact measurement performed in the clinical setting where the analog output is used, since the analog output is proportional to the measured oscillation frequency and does not rely on any assumption from the resonant network. The noise contribution to the measured oscillation frequency is negligible to the macroscopic capacitance variations induced by the contact with the human skin.

## 6. Conclusions

We presented a novel optical probe for diffuse optics measurements based on flexible PCBs, which integrates an accelerometer and a contact sensor based on capacitive proximity sensing. The unique features of flexible PCBs allow for the possibility of building complex optical probes, which provide a direct signal of the quality of the sensor attachment to the skin and its motion, while offering support for the fiber-holders. Constant monitoring of the probe attachment using the proposed design can be effectively used to guarantee subject safety in high-risk applications, such as optical measurements on infants and can finally foster continuous-monitoring acquisition with NIRS devices without the need of a dedicated operator. The presented design can be customized in terms of probe shape, dimensions and equipped sensors based on the specific application, due to the use of a low-cost, commercially accessible technology, flexible PCBs, and can be easily integrated into preexisting measurement setups thanks to analog outputs, USB communication, and battery-powered operation.

## 7. Patents

A patent on the presented contact sensor has been filed by the authors.

## Data Availability

Data underlying the results presented in this paper are not publicly available at this time but may be obtained from the authors upon reasonable request.
